# 10058-F4, a c-Myc inhibitor, markedly increases valproic acid-induced cell death in Jurkat and CCRF-CEM T-lymphoblastic leukemia cells

**DOI:** 10.3892/ol.2014.2277

**Published:** 2014-06-24

**Authors:** QITIAN MU, QIULING MA, SHASHA LU, TING ZHANG, MENGXIA YU, XIN HUANG, JIAN CHEN, JIE JIN

**Affiliations:** 1 Department of Hematology, Institute of Hematology, The First Affiliated Hospital, Zhejiang University School of Medicine, Zhejiang 310003, P.R. China; 2 Key Laboratory of Hematopoietic Malignancies of Zhejiang Province, Hangzhou, Zhejiang 310003, P.R. China; 3 Laboratory of Stem Cell Transplantation, Ningbo First Hospital, Ningbo, Zhejiang 315010, P.R. China

**Keywords:** Jurkat, CCRF-CEM, valproic acid, c-Myc inhibitors, T-cell acute lymphoblastic leukemia, cell death

## Abstract

Adult T-cell acute lymphoblastic leukemia (T-ALL) has a poor prognosis. Although it has been found that activation of Notch1 signaling occurs in >50% T-ALL patients, γ-secretase inhibitors that target Notch1 signaling are of limited efficacy. However, c-Myc is an important direct target of Notch1 and, thus, c-Myc is another potential therapeutic target for T-ALL. Valproic acid (VPA), a histone deacetylase inhibitor, has been reported to treat various hematological malignancies. In the present study, we showed that c-Myc expression, at a transcriptional level, was dose-dependently downregulated in VPA-induced growth inhibition in T-ALL cell lines, Jurkat and CCRF-CEM cells. 10058-F4, a small molecule c-Myc inhibitor, could increase the downregulation of c-Myc and markedly increase the growth inhibition and cell death induced by VPA in Jurkat and CCRF-CEM cells, which was accompanied by obvious cleavage of capase-3. Z-VAD-FMK, a caspase inhibitor, partially prevented the anti-leukemic effect. The results of the present study suggest that c-Myc inhibitors increase cell death induced by VPA in a caspase-dependent and -independent manner, and their combination could be a potent therapeutic strategy for adult T-ALL patients.

## Introduction

T-cell acute lymphoblastic leukemia (T-ALL), an aggressive malignancy arising from T-cell progenitors, accounts for ~15% of ALL cases in children and ~25% in adults. The long-term survival rate for children and adolescents with this disease is 70–75%, while for adults the rate is only 35–40% in Western populations (1). Over 50% of patients with T-ALL harbor activation mutations of Notch1 (2), thus, γ-secretase inhibitors, which block Notch signaling, were a promising target for therapy. However, associated problems, such as limited therapeutic benefit and intestinal toxicity have since been reported (3). Therefore, there is a requirement for the development of novel and effective treatment strategies for adult T-ALL.

Notch1 mutations mainly involve the heterodimerization domain (HD) and the proline, glutamic acid, serine, threonine-rich domain (PEST) (2). The HD mutation results in ligand-independent proteolytic cleavage of Notch1, while the PEST mutation blocks Fbw7 interaction with Notch1 and, thereby, prevents its polyubiquitination and degradation (3,4). The mutations of these two domains result in constitutive activation of the Notch signaling pathway (1). Moreover, ~30% of T-ALL patients harbor inactivating mutations of the Fbw7 gene, which also activate Notch1 signaling (1). It has been demonstrated that c-Myc is a direct and important target gene of Notch1, and its expression levels have been observed to increase along with activation of Notch1 signaling in T-ALL (5). In addition, a previous study showed that the half-life of c-Myc in B-cell or T-cell ALL was markedly prolonged (6). Therefore, c-Myc may be a target for therapy in T-ALL.

Valproic acid (VPA), a histone deacetylase inhibitor (HDACI), has been reported to treat various hematological malignancies (7–9). Previous studies revealed that VPA was more efficacious in combination with other agents, such as idarubicin, 5-azacitidina and all-trans retinoic acid (10–12). In acute myeloid leukemia (AML), VPA inhibited cell growth, mainly by downregulation of c-Myc expression (13). However, the role of c-Myc in the growth inhibition of T-ALL cells induced by VPA remains unclear. We hypothesized that its role in T-ALL was the same as that in AML, and that a c-Myc inhibitor would be able to augment the anti-leukemic effect of VPA. In the present study, the effect of VPA combined with a c-Myc inhibitor (10058-F4) on T-ALL cell lines (Jurkat and CCRF-CEM cells) was investigated.

## Materials and methods

### Cell lines and reagents

T-ALL cell lines, Jurkat and CCRF-CEM cells, were purchased from the American Type Culture Collection (Manassas, VA, USA). These cells were maintained in RPMI-1640 (Gibco-BRL, Grand Island, NY, USA) supplemented with heat-inactivated fetal bovine serum (Gibco-BRL) at 37°C in a 5% CO_2_ humidified incubator. VPA and 10058-F4 (Sigma-Aldrich, St. Louis, MO, USA) were dissolved in dimethylsulfoxide (DMSO) at 1 M and 20 mM and then stored at −20°C in small aliquots. Z-VAD-FMK (Sigma-Aldrich), a pan-caspase inhibitor (14), was also dissolved in DMSO and its final concentration was 20 μM.

### Cell viability assay

Cell viability was measured by 3-(4,5-dimethylthiazol-2-yl)-2,5-diphenyltetrazolium bromide (MTT; Sigma-Aldrich) assay. Briefly, cells were seeded in 96-well plates and treated with VPA (0–3.2 mM), alone or in combination with 60 μM 10058-F4, for 24 h. In each well, MTT solution (final concentration, 0.5 mg/ml) was added and the cells were then incubated at 37°C for 4 h. The absorbance value of each well was measured by a spectrophotometry at 570 nm.

### Flow cytometry

Cell death was detected by Annexin V-fluorescein isothiocyanate (FITC) and propidium iodide (PI) (BD Pharmingen, San Diego, CA, USA) staining. Cells were treated with VPA (0–2.4 mM), with or without 60 μM 10058-F4, for 24 h. Cells were collected in a tube using pipet tips, washed twice in 4°C PBS and then resuspended in 50 μl Annexin V binding buffer (BD Pharmingen). Subsequently, 5 μl Annexin V-FITC and 5 μl PI were added, and these samples were incubated in the dark at 25°C for 15 min. Following the addition of 450 μl Annexin V binding buffer, cell death in the samples was measured on a FACScanto™ II flow cytometer (Becton Dickinson, Franklin Lakes, NJ, USA).

### Quantitative polymerase chain reaction (qPCR)

The expression level of the c-MYC gene was determined using reverse transcription-qPCR with SYBR Green I [Takara Biotechnology (Dalian) Co., Ltd., Dalian, China], and GAPDH was used as a reference gene. Total RNA was extracted using TRIzol (Invitrogen Life Technologies, Carlsbad, CA, USA) and reverse transcribed into cDNA by a SuperScript II first-strand cDNA synthesis kit (Invitrogen Life Technologies). The reaction system contained 12.5 μl 2X SYBR Premix Ex Taq [Takara Biotechnology (Dalian) Co., Ltd.], 1 μl cDNAs and 10 pmol of each primer. The primer sequences were as follows: Forward, 5′-ATGGGGAAGGTGAAGGTCG-3′ and reverse, 5′-GGGTCATTGATGGCAACAATATC-3′ for GAPDH; and forward, 5′-CGTCTCCACACATCAGCACAA-3′ and reverse, 5′-CACTGTCCAACTTGACCCTCTTG-3′ for c-MYC. The reaction was performed at 95°C for 1 min, followed by 40 cycles of denaturation at 95°C for 15 sec and annealing/extension at 60°C for 60 sec, on an iQ5 Real-Time PCR instrument (Bio-Rad Laboratories, Inc., Hercules, CA, USA).

### Western blot

Jurkat and CCRF-CEM cells were lysed at 0°C in lysis buffer for 30 min, and their protein concentrations were measured by the Bradford protein assay method. The samples were separated by 12% sodium dodecyl sulfatepolyacrylamide gel electrophoresis and then transferred onto polyvinylidene fluoride (PVDF) membranes. PVDF membranes were blocked with Tris-buffered saline containing 0.1% Tween (TBST; prepared in the laboratory, Department of Hematology, Institute of Hematology, The First Affiliated Hospital, Zhejiang University School of Medicine, Hangzhou, China) and 5% non-fat milk for 2 h at room temperature. The membranes were then incubated with the primary antibodies, rabbit anti-human monoclonal c-Myc (D84C12 XP^®^), rabbit anti-human monoclonal caspase-3 (8G10) and mouse anti-human monoclonal β-actin (8H10D10) (dilution, 1:1000–5000; Cell Signaling Technology, Beverly, MA, USA), overnight at 4°C. Following washing four times for 10 min each time with TBST, these PVDF membranes were incubated with the goat anti-rabbit (catalogue number, 7074) or horse anti-mouse (catalogue number, 7076) IgG horseradish peroxidase-conjugated secondary antibodies (dilution, 1:2000; Cell Signaling Technology) for 2 h at room temperature. The membranes were again washed four times with TBST, and protein bands were then visualized with the enhanced chemiluminescence detecting kit (EZ-ECL chemiluminescence detection kit; Biological Industries, Beit-Haemek, Israel) and exposed to X-ray films.

### Statistical analysis

The significant differences between experimental and control groups were compared by Student’s t-test. P<0.05 was considered to indicate a statistically significant difference. All statistical analyses were performed using SPSS 16.0 software (SPSS Inc., Chicago, IL, USA).

## Results

### VPA downregulates the expression of c-Myc in Jurkat and CCRF-CEM cells

**Figure 1:**
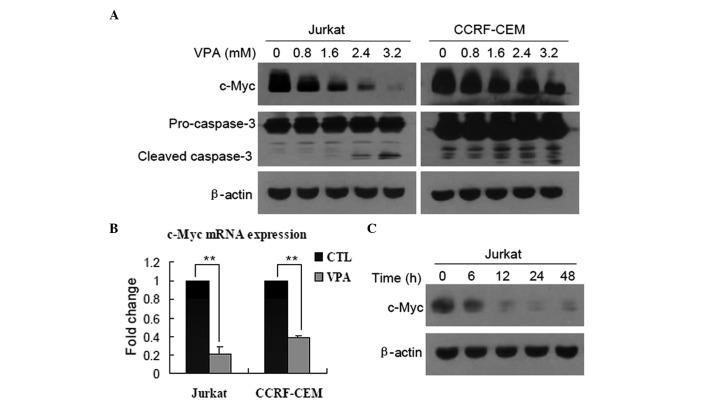
Influence of VPA on the expression levels of c-Myc. (A) Level of c-Myc protein varied in Jurkat and CCRF-CEM cells exposed to 0.8, 1.6, 2.4 and 3.2 mM VPA. (B) Level of c-Myc mRNA varied in Jurkat and CCRF-CEM cells exposed to 0.8 mM VPA. (C) Level of c-Myc protein varied in Jurkat cells exposed to 0.8 mM VPA for 6, 12, 24 and 48 h. ^**^P<0.01. VPA, valproic acid.

First, the role of c-Myc in the growth inhibition of T-ALL cells induced by VPA was identified. Western blots revealed that after Jurkat and CCRF-CEM cells were treated with VPA at various concentrations for 24 h, the expression of c-Myc protein was markedly downregulated in a dose-dependent manner, and was accompanied by cleavage of caspase-3 (Fig. 1A). qPCR was then performed to measure the c-Myc mRNA levels, and it was found that the expression of c-Myc was also decreased in Jurkat and CCRF-CEM cells treated with 0.8 mM VPA for 24 h (Fig. 1B; P<0.01), indicating that c-Myc was downregulated at a transcriptional level. In addition, decreased expression of c-Myc protein in a time-dependent manner was observed in Jurkat cells (Fig. 1C), but not examined in CCRF-CEM cells.

### 10058-F4 further promotes downregulation of c-Myc expression by VPA

**Figure 2:**
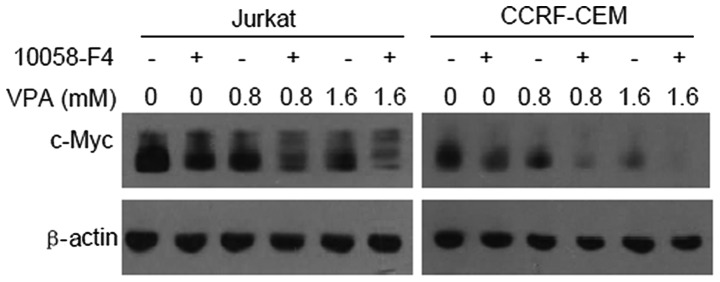
Levels of c-Myc protein in Jurkat and CCRF-CEM cells exposed to VPA combined with 10058-F4 for 24 h. VPA, valproic acid.

It has been demonstrated that 10058-F4 can block the dimerization of c-Myc and Max, and then inhibit c-Myc transactivating activity (15). Thus, we hypothesized that 10058-F4 promotes the downregulation of c-Myc expression induced by VPA. The western blotting results showed that c-Myc expression levels in Jurkat and CCRF-CEM cells treated with VPA (0, 0.8 and 1.6 mM) combined with 60 μM 10058-F4 decreased further compared with the corresponding controls (Fig. 2).

### 10058-F4 increases the growth inhibition of Jurkat and CCRF-CEM cells induced by VPA

**Figure 3:**
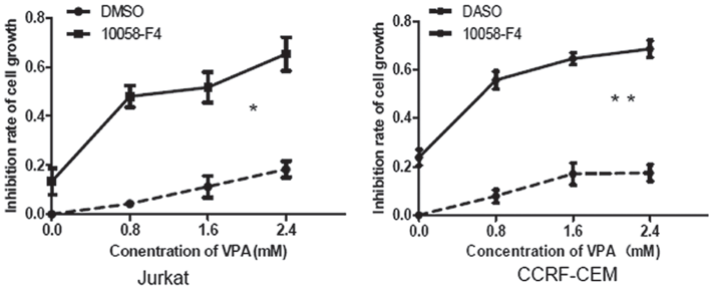
Growth inhibition rates of Jurkat and CCRF-CEM cells exposed to VPA combined with 10058-F4 (60 μM) for 24 h. ^*^P<0.05 and ^**^P<0.01 for all concentrations of VPA. VPA, valproic acid.

c-Myc is an important oncogene, which contributes to the growth of T-ALL cells, particularly T-ALL cells with Notch1 mutations (5). The present study results showed that 10058-F4 and VPA synergistically downregulated c-Myc expression in Jurkat and CCRF-CEM cells. It was next investigated whether 10058-F4 could increase cell growth inhibition induced by VPA in Jurkat and CCRF-CEM cells, by MTT assay. The growth inhibition rates of Jurkat cells treated with VPA (0, 0.8, 1.6 and 2.4 mM) combined with 10058-F4 for 24 h were 17.06±1.03, 47.99±4.35, 51.70±6.19 and 65.27±6.86% respectively, which were significantly higher than those treated with corresponding concentrations of VPA (0, 4.28±0.13, 11.21±4.46 and 17.86±2.60%) (P=0.012). In CCRF-CEM cells, the growth inhibition rates for VPA (0, 0.8, 1.6 and 2.4 mM) plus 10058-F4 were 23.80±3.37, 55.76±3.72, 64.65±2.48 and 68.60±3.48%, respectively, which were also significantly higher than those for the corresponding concentrations of VPA alone (0, 7.92±2.62, 17.03±4.54 and 17.42±3.42%) (P=0.007) (Fig. 3).

### 10058-F4 markedly increases the cell death induced by VPA in a caspase-dependent and -independent manner

**Figure 4:**
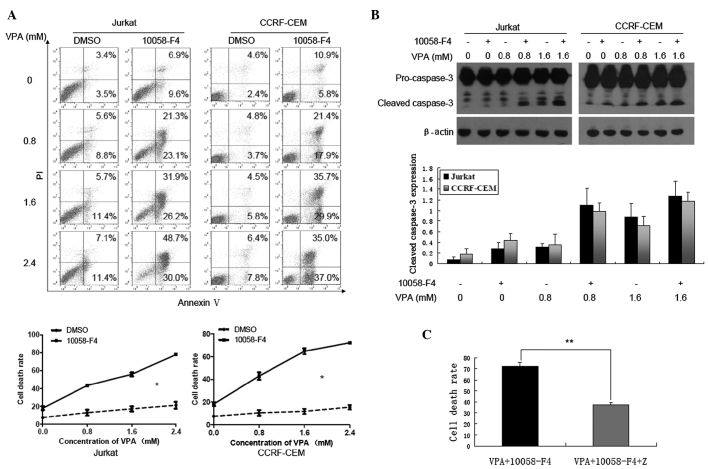
Cell death of Jurkat and CCRF-CEM cells induced by VPA combined with 10058-F4. (A) Cell death was examined by dual staining with Annexin V/PI in Jurkat and CCRF-CEM cells treated with VPA combined with 10058-F4 (60 μM) for 24 h (mean ± SD; n=3). (B) The level of cleaved caspase-3 varied after exposure of cells to VPA combined with 60 μM 10058-F4. Bar graphs were plotted according to the densitometry of the cleaved caspase-3/β-actin band densities (mean ± SD; n=3). (C) Cell death induced by VPA (2.4 mM) combined with 10058-F4 (60 μM) in Jurkat cells was partially inhibited by Z-VAD-FMK (20 μM) (mean ± SD; n=3). ^**^P<0.01 for all concentrations of VPA. VPA, valproic acid; PI, propidium iodide; Z, Z-VAD-FMK.

Previous studies have demonstrated that 10058-F4 efficiently induced cell death in myeloma and AML cells (15,16). Concordant with this, cell death of Jurkat and CCRF-CEM cells treated with VPA (0, 0.8, 1.6 and 2.4 mM) was increased when combined with 10058-F4. As shown in Fig. 4A, compared with treatment with corresponding concentrations of VPA alone, cell death rates (Annexin V^+^/PI^+^ and Annexin V^+^/PI^-^) of Jurkat and CCRF-CEM cells treated with 10058-F4 combined with VPA increased significantly (P=0.038 and P=0.037, respectively). In addition, western blot analysis revealed that 10058-F4 could promote the cleavage of caspase-3 induced by VPA (Fig. 4B). The results also demonstrated that Z-VAD-FMK, a pan-caspase inhibitor, could partially inhibit cell death of Jurkat cells induced by 10058-F4 combined with VPA (72.33±3.35 vs. 37.4±1.87%, P<0.001) (Fig. 4C). These findings indicate that 10058-F4 dramatically increases cell death induced by VPA through caspase-dependent and -independent pathways.

## Discussion

It has been demonstrated that VPA could prevent growth of cancer cells by inducing apoptosis and cell cycle arrest, and by promoting cellular differentiation (17). HDACIs, including VPA, can selectively alter the expression levels of a relatively small proportion of genes by recovering the deacetylation of histones (18). Regarding these genes in which the expression levels are altered by HDACIs, upregulation of the surface TRAIL death receptors (DR4 and DR5) in multiple myeloma (19), upregulation of p21 and p27 in mantle cell lymphoma (20), and downregulation of c-Myc in AML and endometrial cancer cells (13,21) played a key role in the induction of apoptosis and cell cycle arrest or the promotion of cellular differentiation.

Since activating mutations of Notch1 were observed in >50% T-ALL patients (3) and c-Myc is an important direct target of Notch1 (5), the present study examined whether c-Myc was downregulated in the growth inhibition of T-ALL cells induced by VPA. The results showed that, as in AML (13), c-Myc expression was markedly downregulated in Jurkat and CCRF-CEM cells in which activation of Notch1 signaling occurred (22). Further investigation revealed that 10058-F4 could reinforce the downregulation of c-Myc induced by VPA, indicating that the former may increase the sensitivity to the latter.

Previous studies have demonstrated that VPA can induce apoptosis in leukemic cells (7,23). Notably, VPA also augmented apoptosis of leukemic cells induced by other agents, such as cytarabine (24), etoposide (25) and bortezomib (26). Thus, the present study next examined the cell death of Jurkat and CCRF-CEM cells induced by VPA combined with 10058-F4. The results showed that 10058-F4 could markedly increase the cell death of Jurkat and CCRF-CEM cells induced by VPA (0, 0.8, 1.6 and 2.4 mM). Activation of caspase-3 also increased correspondingly. However, Z-VAD-FMK partially inhibited the cell death induced by 10058-F4 combined with VPA, indicating that their apoptotic effects involved in both caspase-dependent and -independent pathways. Previous studies have also demonstrated that not only 10058-F4, but also VPA, induced apoptosis through both caspase-dependent and -independent pathways (15,23,27).

Although there have been arguments for and against the therapeutic targeting of Myc (28), recent experiments *in vivo* and *in vitro* have demonstrated that it is a promising therapeutic strategy in high-risk hematologic malignancies (29,30). The present study showed that downregulation of c-Myc by 10058-F4 markedly increased VPA-induced apoptosis. These findings suggest that VPA combined with c-Myc inhibitors may be a novel potent therapeutic strategy for adult T-ALL patients. However, further investigation with regard to the clinical effect of their combination is required in the future.
